# Social Medical Organizations

**Published:** 1884-03

**Authors:** 


### EDITORIAL NOTES.

### SOCIAL MEDICAL ORGANIZATIONS.

That social intercourse and professional study may be advantageously combined, has been clearly shown in this city by the existence and continued prosperity, during a period of eight years, of the social medical organization, known as the Medical Club The essential features of this society we proceed to state: Its membership is limited to a number that can be conveniently entertained at an ordinary private residence. A unanimous vote is required for the admission of new members, this being considered essential an account of the social element. The meetings are held bi-weekly and consist of the reading of a paper and discussion of the same, followed by clinical reports of interesting cases The meetings are held in routine at the several residences of physicians conveniently located After the adjournment of the meeting proper, social intercourse including a modest collation, completes the evening.

The history of the Medical Club shows not alone that scientific work and social relaxation can be conjoined, but proves the advantage of the combination. The meetings of the Medical Club are attended by an average of two-thirds of its membership. It has done more to promote medical thought and progress than all the organizations in the city and county combined. In addition to all this, and of perhaps equal importance, has been the working of the social element in softening the asperities, and counteracting the many causes of jealousy, among medical men. Through its agency the members have learned to respect the many good qualities of heart and mind of their associates, and in turn have been prompted to renewed endeavors and the establishment of a higher standard for themselves.

It has been sagely remarked that imitation is the highest praise, and certainly duplication indicates most earnest endorsement. For reasons already stated, we hail with sincere pleasure the establishment within the past three years of the Medical Union, an organization of similar constitution and purposes with the Medical Club. The well written article on Dysentery, in the preceding number of the JOURNAL, was prepared and read by a member of this more recently established Social Medical Organization. Its membership is already practically completed, showing, as we believe, that the field is still open for similar coteries. Of the Medical Union we anticipate beneficent influences no less marked than those shown by the past history and expected from the future of the Medical Club To its members the editors of the JOURNAL wish much scientific improvement as well as social pleasure, and it is their hope that the pages of the JOURNAL may, from time to time, be enriched with the most interesting and best digested of their clinical reports and original papers.

